# Depictions of older adults residing in nursing homes: a Goffman-inspired thematic analysis of children’s picture books in a Danish context

**DOI:** 10.3389/fsoc.2025.1588854

**Published:** 2025-05-30

**Authors:** Stinne Glasdam, Ragnhild Julante Andersen Gulestø, Christie Stilwell

**Affiliations:** ^1^Health Sciences, Lund University, Lund, Sweden; ^2^Department of Health Sciences, Institute of Nursing, VID Specialized University, Oslo, Norway; ^3^Faculty of Health, Dalhousie University, Halifax, NS, Canada

**Keywords:** children’s picture books, Denmark, Goffman, nursing home, older adults

## Abstract

**Introduction:**

Some children may witness their grandparents or great-grandparents spending their final days in a nursing home, while others have peers with older relatives living in nursing homes. Children learn about ageing through experience and exposure, where picture books can also shape their perceptions and attitudes toward older adults residing in nursing homes.

**Aim:**

To explore the underlying beliefs and attitudes about ageing and older adults residing in nursing homes depicted in children’s picture books in a Danish context.

**Methods:**

A systematic search of children’s picture books was conducted in the national Danish library catalogue. The search strategy initially identified 360 books, all were manually screened against the inclusion and exclusion criteria. In total, 22 met the inclusion criteria. Data were extracted and analysed using a descriptive and reflexive thematic analysis, methodologically inspired by Braun and Clarke and theoretically informed by Goffman.

**Results:**

The empirical material consisted of 439 pages containing text and/or illustrations depicting nursing homes and/or older adults living in them. The first editions were published between 1973 and 2022, with all books (re)printed in Denmark after 1975. The books originated from nine countries, with the majority from Denmark (*n* = 9). Three overarching themes were constructed: “*Nursing homes—a housing solution between necessity and coercion*,” “*Associations between nursing homes and kindergartens*,” and “*The present was the past and death was the future*.” Nursing homes were depicted both as care facilities and places of forced residence, often akin to kindergartens and reinforcing the infantilization of older adults. Older adult characters were often depicted as equals to children, while the middle generation functioned as a bridge between generations without much interaction with older adults. Few books addressed death directly, and those that did tended to portray it in romanticized terms, surrounded by a nuclear family.

**Conclusion:**

Picture books reinforced certain stereotypes about nursing homes, older adults, and family dynamics. Future publications should consider ways to integrate realistic and diverse depictions of nursing homes, their residents, and friends and family that visit.

## Introduction

In Western societies, older age is often associated with frailty, illness, and dependency (Olaison et al., 2022; Strand et al., 2023). This can lead to greater reliance on healthcare services and supports (Almevall et al., 2022) and may eventually necessitate moving into a nursing home. However, ageing consists of biological, psychological and social processes and a social construct shaped by cultural representations, institutional practices, and policy regimes (Blix and Ågotnes, 2023). As critiques of the “successful ageing” paradigm have shown (Blix and Ågotnes, 2023; Rubinstein and de Medeiros, 2015), cultural discourses that emphasise individual responsibility in later life often reflect broader neoliberal logics. One such example is the increasing emphasis on “ageing in place” strategies, which focus on enabling older adults to remain in their own homes or familiar environments for as long as possible (Alders and Schut, 2019; Buffel and Phillipson, 2024; Rubinstein and de Medeiros, 2015). These strategies often lead to fewer nursing home placements, while also acknowledging that nursing homes are essential and that such facilities are necessary only for those with high care needs (Finlay and Finn, 2021; Pasveer et al., 2020; Synnes and Frank, 2020). In Denmark, for instance, between 2010 and 2021, the number of people aged 75 and older living in nursing homes and senior housing decreased by 11%, even as the population in that age group grew by 41% (Danmarks Statistik, 2021). As a result, the percentage of the older population living in nursing homes and senior housing has decreased. In 2010, 15% of those aged 75 and older resided in nursing homes or senior housing, compared to 9% in 2021. This decrease is particularly pronounced among individuals aged 90 and above, as in 2010, 42% lived in care facilities, while that number dropped to 32% in 2021 (Danmarks Statistik, 2021). Even though the use of nursing homes is slightly declining, many children may witness their grandparents or great-grandparents spending their final days and passing away in a nursing home. Additionally, children may have peers whose older family members reside in nursing homes.

Children’s socialisation occurs through primary family relationships and broader institutional and cultural exposure (Berger and Luckmann, 1966; Bourdieu, 1996). Children’s perceptions of old age and intergenerational care are shaped by encounters with older adults, including those living in nursing homes, whether these interactions are direct or occur through peers (Crawford, 2015; Newman and Hatton-Yeo, 2008). Many initiatives support children’s socialisation into society and its sub-worlds, such as healthcare systems, including nursing homes. Through these channels, children form impressions of the world, shaping their understanding of various people and social categories, including how they perceive groups such as older adults. In an increasingly age-segregated society, intergenerational programmes have emerged to foster contact between generations. Such initiatives are growing steadily and are shown to help children understand the abilities and limitations of older adults, fostering empathy and emotional awareness (Laging et al., 2022; Wendland and Parizet, 2023). These programmes also serve as forms of socialisation, where children encounter institutional care settings and learn about the social place of older people. However, while these interactions may promote empathy, they can also expose societal ambivalence about ageing and the erosion of intergenerational solidarity under neoliberal restructuring. For example, when institutional care is framed through market logic, as Kramer (2024) notes, children’s exposure to such settings may also reflect neoliberal imaginaries, where care becomes a service, not a collective responsibility. This framing can subtly influence how children come to value ageing and dependency.

Beyond lived experiences, children also encounter ageing through cultural representations, such as in picture books, which often serves as an early and powerful medium of socialisation. These books are not neutral artefacts. They offer narratives that can reinforce or challenge prevailing ideologies. Picture books integrate text and image in ways that demand active interpretation (Nodelman, 1988), enabling children to make sense of complex social worlds. In particular, picture books combine visual and textual narratives seamlessly, compared to illustrated storybooks where the text drives the plot, and the images serve as a supporting role (Matulka, 2008; Barder, 1976). In picture books, both elements are essential for understanding the whole message. Nodelman (1988) highlights their dynamic interaction, which is sometimes complementary and sometimes contradictory but prompts deeper engagement. This multimodal format makes picture books powerful tools for meaning-making, especially when addressing complex social topics. Children interpret picture books by synthesising visual and verbal cues (Sipe, 1998, 2011; Nikolajeva and Scott, 2006). While naturalistic illustrations once dominated, Danish picture books have shifted toward more abstract forms since the 1950s (Christensen, 2003). Rather than being passive consumers, children actively engage with these texts (Jenkins, 1992), which can help them process topics such as illness, bereavement, and loss (Doering, 2021; Wiseman, 2013). Studies show that picture books can serve therapeutic and educational functions by assisting children in navigating difficult emotions and events. They can facilitate children’s understanding of personal circumstances by presenting relatable scenarios within the safe confines of a story (Doering, 2021). Additionally, like films (Sgier et al., 2024) and storybooks (Martínez-Caballero et al., 2023), picture books have proven effective in helping children to process death and grief (McGuire et al., 2013; Wiseman, 2013). By addressing such topics, picture books not only support social and emotional development but also provide children with tools to interpret complex social dynamics and challenging experiences. Additionally, they may contribute to the symbolic construction of ageing and older adults by depicting what it means to grow old, and where.

Through the dynamic interplay of text and image, picture books encourage children to engage actively with content, fostering a deeper understanding of themselves and the world around them. Given that picture books often serve as a child’s first cultural introduction to different stages of life, including later life, analysis of how institutional care and older adults are depicted offers insight into how children are socialised into dominant or resistant discourses about age, dependency, and care. By examining the visual and narrative representations of older adults living in nursing homes in Danish children’s picture books, we can better understand the messages children are receiving about ageing, dependence, and the roles older adults play within families and communities. It is therefore important to examine how children’s picture books contribute to the socialisation of young readers by shaping their understanding of older adults living in nursing homes. These representations can either reinforce or challenge ageist tropes. By focusing on how ageing, institutional care, and intergenerational relationships are constructed in these texts, we highlight the visual and narrative portrayals that reveal underlying assumptions and perceptions about older adults residing in such facilities. Therefore, this study aims to explore the underlying beliefs and attitudes about ageing and older adults in nursing homes depicted in children’s picture books in a Danish context.

## Materials and methods

This study was a review of children’s picture books intended for readers aged 3–8 within a Danish context, focusing on older adults living in nursing homes. A thematic analysis of the included picture books was conducted, methodologically inspired by Braun and Clarke (2006) and Braun et al. (2019) and theoretically informed by Goffman (1955, 1990). The analysis examined how images and text collectively portray constructs and messages about nurses and nursing, both textually, visually, and contextually (Azano et al., 2017; Sipe, 1998, 2011).

### Theoretical framework

Erving Goffman (1955, 1990) drew on metaphors from theatre and dramaturgy to analyse individuals’ behaviours and interactions, illuminating the values, norms, and beliefs that shape societies. He depicted social life as a stage, where individuals are cast into roles that they must perform, learning from others how to enact these roles through socialisation. On this stage, individuals interact by presenting themselves to others, with everyone simultaneously performing and observing others’ performances. Goffman conceptualised this by using terms like “front stage,” “backstage,” “performance,” “roles,” “framing,” and “audience” (Goffman, 1955, 1990).

In his theory of interaction, Goffman (1990) examined front-stage performances, where individuals engage in self-presentation, governed by socially prescribed rules and conventions. Here, performers interact with an audience that observes, evaluates, and reacts, influencing how the performance is received. Such performances help individuals project a coherent self-image, striving to “save face” and maintain their social standing. Backstage, however, individuals are away from the public eye, allowing them to relax, express their genuine emotions, and reflect on their experiences, which then shape their public behaviour. Rituals, norms, and societal expectations further dictate how these front and backstage performances unfold (Goffman, 1990). While audiences often perceive front-stage performances as authentic and credible, they may also question the performers’ sincerity and competence. Within different contexts, individuals have defined roles and statuses and work together to achieve common goals. The social environment, or “frame,” represents the physical space where interactions and performances occur, with varying degrees of formality depending on the situation (Goffman, 1990).

This study examined children’s picture books to analyse how older adults were depicted in their roles, interactions, and performances across different settings. These included private spaces such as individual apartments within nursing homes, and common areas like living rooms, communal dining areas, and outdoor spaces. We analysed how the different actors in the picture books performed either individually or collaboratively across various contexts and also examined who was positioned as the audience in the different situations depicted. Using Goffman’s dramaturgical framework, this study investigated how these narratives portrayed older adults’ lives, social interactions, and self-presentations across various settings within nursing homes, while also examining which norms and rituals were present.

### Data collection

The systematic data collection process followed specific inclusion and exclusion criteria to ensure the relevance and accessibility of selected materials. The inclusion criteria required that each book met the following parameters: (1) children’s picture book targeting young children 3–8 years of age, (2) available in printed or electronic formats at Danish Libraries, (3) written in or translated into Danish, (4) addressed themes of nursing homes and older adults living in nursing homes—either as a central focus or incorporated into broader narratives, and (5) was originally published or reprinted between 1975 and 2024. We selected a 50-year period partly because there were not many books on the subject and partly because all these books were available in Danish children’s libraries. Exclusion criteria were song books, rhymes, and rhyming verses.

First, a test search was conducted in the local Odense library catalogue^1^ with the help of a librarian using the following search terms: (plejehjem* OR aldersdomhjem*) AND (billedbog OR billedbøger). In English, this translates to nursing home or retirement home and picture book(s). It resulted in only 12 hits, therefore an advanced search for children’s picture books was conducted in the national Danish library catalogue^2^ using the following search terms: (plejehjem* OR aldersdomhjem* OR “ældre mennesker” OR “gamle mennesker” OR *mor OR *far OR *foræld* OR død*). In English, this translates to (nursing home OR retirement home OR old* people OR elderly people OR *ma/mother OR *dad/father OR *parent* OR dead). The asterisks (*) are used as a wildcard character to capture one or more characters in a search term, for example the term *mor will include related terms such as *farmor*, *mormor*, *bedstemor*, and *oldemor* (Danish terms for grandma and great-grandma). The search was limited to picture books (billedbog OR billedbøger) written in or translated into Danish, published within the last 50 years, specifically from 1975 to 2024. The search was conducted between November 2024 and January 2025, with the final search completed on 2 January 2025. The initial search retrieved a total of 360 books, from which 5 duplicates were removed. All identified books were manually screened against the inclusion and exclusion criteria in a collaborative process among two of the authors (SG and CS). The third author (RJAG) participated in the assessments in a few cases of doubt. In total, 333 books were excluded because they did not meet the inclusion criteria: 318 were unrelated to the topic, nine were song books with rhymes or rhyming verses, five were for readers aged 9 and older, and one was published before 1975 and had not been reprinted. All books were available in the Danish libraries’ collections or as electronic copies. Ultimately, 22 books met all criteria and were included in the analysis.

### Analysis strategy

This study’s analysis of children’s picture books examined how illustrations and text work together to convey themes of older adults in nursing homes to young readers in Denmark, considering both visual/textual presentation and contextual framing (Azano et al., 2017; Sipe, 1998, 2011). The analysis comprised two parts: (1) a descriptive numerical summary titled “Characteristics of the Included Books” and (2) a reflective thematic analysis. Methodologically, the reflexive thematic analysis was inspired by Braun and Clarke (2006) and Braun et al. (2019) and theoretically informed by the concepts of Goffman (1955, 1990).

First, all included books were read several times by all three authors, aiding in familiarisation with the material (Braun and Clarke, 2006). Information gathered from each book included the author (and translator, if applicable), illustrator, publication year, title in Danish (original title if different), country of origin, and general characteristics. Relevant images and text passages related to older adults in nursing homes were then extracted and saved in a digital database. For the analysis, only sections specifically addressing older adults in nursing homes in line with the study’s objectives were included. The extracts were organised by using analytical questions inspired by Goffman (1955, 1990), which served as coding prompts to categorise and interpret the content of the picture books. The initial analysis focused on the following Goffman-inspired questions, examining how children’s book authors portrayed scenes featuring older adults in nursing homes:

- In which locations or settings within nursing homes were older adults depicted?
- What roles did older adults assume, and how were these roles enacted?
- Which costumes and props were used?
- Who were the co-actors and the audience, and how and where did they interact with older adults?

Two of the authors (SG and CS) conducted the initial data extraction based on the questions above, while the third author (RJAG) reviewed all books and data extraction, supplemented, and validated the extraction. Through coding and reorganisation of the empirical material, preliminary themes were developed with an emphasis on identifying both similarities and differences. Inspired by Braun and Clarke (2006) and Braun et al. (2019), these initial themes were critically reflected in an iterative dialogue among the authors, involving discussions that compared the themes with the content of the picture books. This ensured that the themes accurately reflected both the book content and the objectives of the current study. Throughout this process, all authors engaged in continuous discussion, guided by Goffman’s theoretical framework. Refinements to the initial themes ultimately led to the identification of three final themes (see Table 1: “*Nursing homes—a housing solution between necessity and coercion*,” “*Associations between nursing homes and kindergartens*,” and “*The present was the past and death was the future*”).

**Table 1:** Themes and representative examples from the books.

*Nursing homes—a housing solution between necessity and coercion*		
Author (year), Book title	Book excerpt (translated to English)	Element represented in the theme
Aakeson (2022), Eventyr	Text: “I miss the old red house, just with Grandma in it. ‘*What house are we talking about?*’ Grandma asks. Wow, I think, now she’s gotten bad at remembering her own house too. (Dad says) “*Old people get so old that they forget; they can forget everything. Maybe Grandma will forget who we are someday too.*” Picture description: In the common room of the nursing home, 11 older residents are mostly depicted sitting in chairs, though two older men are bent over looking at something on the ground. There is a doctor in the doorway and a younger assistant helping to feed a resident. A variety of mobility aids are shown (e.g., cane, rollator walker, wheelchair). One man eating is wearing a bib. None of the residents are smiling. The main colours are muted tones of dusty blue and beige.	Nursing homes were necessary living arrangements for people with dementia.
Zavrël (1094), Morfar Thomas	Text: “One fine day, the mayor got out of bed on the wrong side. *“All the old people must leave the city and live in a brand-new, big house, ‘Smile House’”* he said. *“Why is that?”* asked the secretary. *“They’ve grown old, so they set a bad example for others. They do not look very nice anymore, they are no longer useful, and they wander around without paying attention to traffic. They give children chocolate and tell them stories in the evening instead of letting them watch television. This cannot go on.”* Picture description: The mayor is shown in a dark office setting surrounded by equipment covered in gears, lights, and dials. He is dressed in a green, military-style uniform and holds a smiling mask in front of his face, covering his stern expression. He is giving orders to three men in lab coats holding nets, who have been tasked with catching the older people. The white lab coats, green uniform, and yellow overhead light draw offer contrast with the dark background.	Nursing homes were presented as solutions for older adults that were perceived as lacking societal value.
Marsoli (2009), Op	Text: “Shortly after [a visit from a ‘wilderness scout’], Carl received bad news. He would be forced to leave his house and move into a care home. Carl did not want to leave his house; after all, it held all his memories of Ellie [his deceased wife].” Picture description: From the vantage of peering over his shoulder, the drawing shows a close-up side profile of Carl’s frowning face while he looks at a black and white photo in his journal. A little, brightly coloured drawing of his beloved house is taped to the corner of the photo. The background of the drawing is muted mauve, which contracts against his white hair and dark framed glasses.	Nursing homes were presented as residential options for older adults whose houses (despite offering resistance) were demolished due to development initiatives.
*Associations between nursing homes and kindergartens*		
Shepherd (2022), Bedstemor	Text: “Dad says it’s important for Grandma to live somewhere she feels safe. She needs to have people around her who—better than we—can help her.” Picture description: Grandmother has curly hair and rosy cheeks. She wears a striped shirt and red pants. Her helpers wear yellow and grey scrubs. Colours are bright and bold, and simply illustrated, focusing on the characters and less on the background.	Nursing homes depicted as care settings where older individuals were ‘dropped off’ by the middle-aged generation (adult children).
Steiner (1976), Hvordan er det at være… GAMMEL	Text: “Aunt Kirsten likes to make nice things. Not everyone who is 88 years old can still make things. Most people stop working when they get old. They’ve worked so much that they become tired and worn out. Then it’s nice to stop working. Maybe they do something completely different, like reading books, sewing, or weaving. Taking care of their garden. Or they might go to a kind of school because they want to learn something new.” Picture description: A black and white photograph of Anna and her Aunt sitting at a table with a checkered tablecloth working a craft together. Anna wears a plaid dress; Aunt Kirsten wears a wool overcoat. The craft is a tree sculpture. Aunt Kirsten is reaching for a tool, and Anna holds a stick with a ball on the end.	Older adults adjusted their behaviour based on their social context or setting and became more playful and engaged with recreational activities similar to kindergarten.
Wagelin-Challis (2007), Vi tænker på dig, farfar!	Text: “My grandfather is very old, almost 100 years. He walks incredibly slowly, but whenever we are on our way to the dining hall, he always wants us to race to see who gets there first.” Picture description: Grandfather is quickly pushing a walker down the hall while his two grandchildren run around him. Grandfather wears a fedora, glasses, long brown slippers, and grey clothes, with money and a tie hanging out of his back pocket and his pants tucked into his socks. He has a wispy moustache, hairs that stick out above his ears, and a smile. His granddaughter wears all pink and his grandson wears all green.	Children/intergenerational connections promoted playful behaviours and activities among older adults.
*The present was the past and death was the future*		
Dreesen (2006), Mormor på sommereng	Text: Grandma turns to Esther, “You do not say much, huh? Are you shy?” Grandma does not know she’s a grandma anymore. Picture description: Esther, her mother, and Grandma sit at a table. Her mother and Grandma have coffee. Grandma is wearing a bright blue shirt, patterned blue skirt, with short grey hair and a smiling expression. Esther is wearing a white dress and has a stuffed toy on her lap. Mother is wearing a red and white striped shirt. The illustration style is slightly abstract. The characters stand out against the plain background.	Older adults with cognitive decline were shown as not connecting to the present and forgetting the past. They were ‘absent’ even when they were physically present.
Haller (2021), Farvel olde, en bog om døden, afsked og minder	Text: “As they enter the room, Great-grandmother is lying with her eyes closed, but she opens them slightly and smiles a little when she hears them.” Picture description: Great-grandmother is laying in a hospital-style bed with a colourful quilt draped over the end. There are framed pictures of her wedding, her children and grandchildren on the wall. She is surrounded by family members. She is touching fingers with her grandson, and a picture drawn by the grandchildren is on the bed. The colours of the room and clothing of the family are bright and cheerful, and one family member has a subtle tear on their face.	The act of dying was swift, and passing was peaceful.
Moen (1988), Gammelmor og Tullemor	Text: “Most of the old adults were in the living room. Some were sitting in a rocking chair. They rocked and slept. Some just sat there staring into space. They hardly moved. The only one moving was an old man, who paced back and forth all the time. Grandma sat in her chair in her room. There she sat—alone with herself. And she was sad.” Picture description: Gammelmor is sitting in a chair beside a window. She has white hair, and her eyes are barely open. She wears a blue dress, dotted grey shawl, and dark shoes and stockings. Her expression is sad and defeated. Her glasses and a half drank beverage sit on the windowsill. The colours of clothes and the food and walls are muted and gloomy.	Nursing homes were waiting rooms for death: depicted through loneliness, silence, stillness, and darkness.

As the authors of this article did not seek permission from the picture books’ original authors to publish images, the results section presents detailed narrative descriptions of the selected illustrations instead.

## Findings

### Characteristics of the included picture books

The 22 included picture books, as shown in Table 2, comprised a total of 439 pages featuring text and/or illustrations of nursing homes or/and older adults living in nursing homes. The first editions of these books were published between 1973 and 2022, and all were (re)printed after 1975 in a Danish context and available at Danish public libraries. Of these books, two were written in the 1970s, four in the 1980s, one in the 1990s, five in the 2000s, six in the 2010s and four in the 2020s. These books originated from Denmark (*n* = 9), Sweden (*n* = 4), the United States of America (*n* = 2), Norway (*n* = 2), Germany (*n* = 1), Belgium (*n* = 1), Australia (*n* = 1), Switzerland (*n* = 1), and the Netherlands (1).

**Table 2:** The included picture books.

Author (translator)	Book title in Danish (Original title) [English translation]	Publication year of Danish version (Original country; year)	Illustrator
Aakeson, Kim Fupz	Ting der bliver væk (Ting der bliver væk) [Things that get lost]	2021 (Denmark; 2021)	Hole, Stian
Aakeson, Kim Fupz	Eventyr (Eventyr) [Adventures]	2022 (Denmark; 2022)	Hole, Stian
Abel, Cecilia Hafezan	Nu er det min tur - Roberta er besøgsven (Nu er det min tur - Roberta er besøgsven) [Now it’s my turn—Roberta is a visiting friend]	2015 (Denmark, 2015)	Abel, Cecilia Hafezan
Amant, Kathleen (Jensen, Gry Kappel)	Anna besøger plejehjemmet (Anna in het bejaardentehuis) [Anna visits the nursing home]	2018 (The Netherlands; 2018)	Amant, Kathleen
Bourgeat, Pierre	Osteklokken (Osteklokken) [The Bell Jar]	1984 (Denmark; 1984)	Bourgeat, Lene
Dreesen, Jaak (Jørgensen, Mette)	Mormor på sommereng (Oma in de zomerwei) [Grandmother on a Summer Meadow]	2006 (Belgium; 2005)	Westerduin, Anne
Fox, Mem (Nielsen, Carl)	Morten Storbjørn Alexander Hansen (Wilfrid Gordon McDonald Partridge) [Wilfrid Gordon McDonald Partridge]	1986 (Australia; 1984)	Vivas, Julia
Haller, Ina Victoria	Farvel, Olde - En bog om døden, afsked og minder (Farvel, Olde - En bog om døden, afsked og minder) [Farewell, Olde - A book about death, farewell and memories]	2021 (Denmark; 2021)	Letén, Lea
Lundgren, Max (Albert, Jørn E.)	Jespers farfar (Mats farfar) [Jesper’s grandpa]	1979 (Sweden; 1970)	Hald, Fibben
Madsen, Jacinta	Leg på plejehjem - da Lea mødte Rosa (Leg på plejehjem - da Lea mødte Rosa) [Play at a nursing home - when Lea met Rosa]	2020 (Denmark; 2020)	Madsen, Jacinta
Marsoli, Lisa (Holm, Dorte)	Op (Up) [Up]	2009 (United States of America; 2009)	Egan, Caroline
Moen, Thorhild (Leth, Wivi)	Gammelmor og Tullemor (Gamlemor og Tullemor) [Grandma and Tullemor]	1988 (Norway; 1987)	Scheen, Kjersti
Nivaa, Claus	Mormors taske (Mormors taske) [Grandma’s handbag]	2018 (Denmark; 2018)	Bowyer, Martin
Preuss, Mathias (Frederiksen, Carsten)	Gorm og de gamle (Paul und die seltsamen Leute im Altenheim) [Gorm and the elderly]	2005 (Germany; 2005)	Preuss, Mathias
Rosenbeck, Anna and Thormann, Inger	Da Martins bedstefar døde (Da Martins bedstefar døde) [When Martin’s grandfather died]	2009 (Denmark; 2009)	Seeberg, Ursula
Rørvik, Bjørn F. (Gyldenkærne, Nanna)	De tre Bukke Bruse vender tilbage (Bukkene-Bruse vender tilbake) [The Three Billy Goats Gruff Return]	2015 (Norway; 2014)	Moursund, Gry
Scherfig, Lilja	Farfar (Farfar) [Grandpa]	2015 (Denmark; 2015)	Dickmeiss, Otto
Shepherd, Jessica (Andersen. Anja)	Bedstemor (Grandma) [Grandma]	2022 (United States of America; 2014)	Shepherd, Jessica
Stark, Ulf (Hartmann, Nils)	Kan du fløjte, Sofie (Kan du vissla Johanna) [Can you whistle, Sofie?]	1993 (Sweden; 1992)	Höglund, Anna
Steiner, Marie-Louise (Ib Permin)	Hvordan er det at være…. GAMMEL (Är det så att bli gammal) [How is it to be… OLD]	1976 (Sweden; 1973)	Steiner, Marie-Louise
Wagelin-Challis, Sven (Bodenhoff, K)	Vi tænker på dig, farfar! (Vi tänker på dig, farfar!) [We’re thinking of you, Grandpa!]	2007 (Sweden; 2007)	Garhamn, Anna-Karin
Zavrël, Stêpan (Glistrup, Eva)	Morfar Thomas (Grossvater Thomas) [Grandfather Thomas]	1984 (Switzerland; 1983)	Zavrël, Stêpan

All the books have both children and older adults as the main protagonists. Of these, 12 of the children are boys and nine are girls; in one book, both boys and girls are main protagonists (Zavrël, 1094). In 11 of the books, the older adult is a man, and in 10 of the books, a woman; one book has both men and women as main protagonists (Bourgeat, 1984). The children and older adult main protagonists had the same sex in 16 books and different sex in three books (Amant, 2018; Fox, 1986; Shepherd, 2022). Sixteen books focused on the children’s visit at the nursing home (e.g., visiting their grandparents) (Aakeson, 2021, 2022; Amant, 2018; Dreesen, 2006; Lundgren, 1979; Madsen, 2020; Moen, 1988; Nivaa, 2018; Preuss, 2005; Shepherd, 2022; Stark, 1993; Steiner, 1976; Wagelin-Challis, 2007), visiting friends (Abel, 2015; Fox, 1986) and participating in intergenerational projects (Madsen, 2020). Eight books focused on older adults with dementia and their relationships with their children from the perspective of their grandchildren (Aakeson, 2021, 2022; Dreesen, 2006; Fox, 1986; Lundgren, 1979; Moen, 1988; Nivaa, 2018; Shepherd, 2022). Three books described older adults and children as each other’s lifeline for involuntary living conditions (Bourgeat, 1984; Rørvik, 2015; Zavrël, 1094). The death of older adults was depicted in three books (Haller, 2021; Rosenbeck and Thormann, 2009; Stark, 1993). The remaining books focused on other topics such as friends living abroad and how the sense of longing sparks reflections on loss (Aakeson, 2021); death, bereavement and memories (Haller, 2021); ageing and lives as older people (Steiner, 1976); a new-interpretation of the fairy tale “The three Billy Goats” (Rørvik, 2015); the life story of an old man who lost his spouse and refuses to move from his home (Marsoli, 2009); and an old man forced to live with his family as the nursing home not have a room him (Scherfig, 2015); but all have story elements related to older adults living in nursing homes.

The results focused on nursing homes as a backdrop for the stories, functioning both as backstage and front stage areas in the depiction of older adults living there. Several picture books portrayed nursing home buildings and their surroundings. Some nursing homes were depicted as small castles (Dreesen, 2006; Madsen, 2020; Rørvik, 2015; Shepherd, 2022), a villa (Fox, 1986; Stark, 1993), and a prison-like building (Zavrël, 1094). Some nursing homes were located close to the church and the cemetery (Stark, 1993), others were in park-like surroundings (Dreesen, 2006; Madsen, 2020; Rørvik, 2015; Shepherd, 2022), or residential neighbourhoods (Fox, 1986), and others were located in a sinister area outside the city (Zavrël, 1094). In the picture books, some of the nursing homes had a garden (Dreesen, 2006; Lundgren, 1979; Madsen, 2020; Rørvik, 2015; Shepherd, 2022). Two of the nursing homes had names, called “The house of smiles” (Zavrël, 1094) and “The tranquillity of the forest” (Rørvik, 2015).

The interior of the nursing homes was depicted in various ways in the picture books. In Shepherd (2022) picture book, there is a black-and-white floor plan of a section of the nursing home, including corridors, reception, two older adult’s rooms, elevators, stairs, the dining room, and the living room. Some picture books portrayed nursing homes with long corridors lined with adjoining rooms, each behind a door housing an older man or woman (Abel, 2015; Amant, 2018; Rørvik, 2015; Stark, 1993; Wagelin-Challis, 2007). Some corridors had windows, others not; some had paintings/photos on the walls, others not; some walls and floors had strong colours, others were grey (Aakeson, 2021; Abel, 2015; Amant, 2018; Dreesen, 2006; Preuss, 2005; Rørvik, 2015; Stark, 1993; Wagelin-Challis, 2007; Zavrël, 1094). In the displayed living rooms, older adults sat in armchairs/dining chairs or wheelchairs, with tables and a few plants. Some walls and floors in the living rooms had strong colours, others had dark, sombre (grey, yellow, brown nuances) colours (Aakeson, 2022; Amant, 2018; Fox, 1986; Moen, 1988; Preuss, 2005; Shepherd, 2022; Stark, 1993; Steiner, 1976). In 15 of the picture books, older adults’ rooms at the nursing homes were depicted, all of them were single rooms, where some walls and floors had strong colours; others were muted colours in shades of white, grey, and yellow (Aakeson, 2021, 2022; Abel, 2015; Amant, 2018; Dreesen, 2006; Haller, 2021; Lundgren, 1979; Moen, 1988; Nivaa, 2018; Preuss, 2005; Rosenbeck and Thormann, 2009; Shepherd, 2022; Stark, 1993; Steiner, 1976; Wagelin-Challis, 2007). Some of the pictures showed the bed of the older adult and assistive devices (Aakeson, 2021; Abel, 2015; Amant, 2018; Haller, 2021; Lundgren, 1979; Preuss, 2005; Rosenbeck and Thormann, 2009; Shepherd, 2022; Stark, 1993; Wagelin-Challis, 2007), others showed a dining table and/or armchairs (Aakeson, 2021, 2022; Amant, 2018; Dreesen, 2006; Lundgren, 1979; Moen, 1988; Nivaa, 2018; Preuss, 2005; Rosenbeck and Thormann, 2009; Shepherd, 2022; Stark, 1993; Steiner, 1976; Wagelin-Challis, 2007), and others showed assorted furniture, lamps, plants, photos and artwork, etc. (Aakeson, 2021; Amant, 2018; Haller, 2021; Lundgren, 1979; Nivaa, 2018; Shepherd, 2022; Stark, 1993; Wagelin-Challis, 2007). In some picture books, other rooms in the nursing homes were also shown such as a big hall (Bourgeat, 1984), activity/exercise room (Amant, 2018; Madsen, 2020), hairdresser (Amant, 2018), canteen (Amant, 2018; Wagelin-Challis, 2007), stairwells and shared dormitory (Zavrël, 1094).

#### Nursing homes—a housing solution between necessity and coercion

Nursing homes were presented in four overarching ways: (1) as necessary living arrangements for solitary older adults who, for various reasons, were unable to live alone and take care of themselves in their daily lives, (2) as places of forced residence, (3) as places older adults escaped from, and (4) as places lacking capacity for all older adults in need. Many of the picture books showed that nursing homes were necessary living arrangements for people with dementia (Aakeson, 2021, 2022; Dreesen, 2006; Fox, 1986; Lundgren, 1979; Moen, 1988; Nivaa, 2018; Shepherd, 2022). Other books presented it as necessary living arrangements for older adults who used wheelchairs and walkers (Aakeson, 2021, 2022; Abel, 2015; Amant, 2018; Dreesen, 2006; Preuss, 2005; Madsen, 2020; Shepherd, 2022; Scherfig, 2015), older adults who were not able to hear (Amant, 2018; Wagelin-Challis, 2007), older adults having health conditions such as heart disease (Steiner, 1976) and eating problems (Preuss, 2005). Some were bedridden due to pain throughout their bodies (Abel, 2015). The older adults were depicted in the role of being in need of help from healthcare professionals who acted front stage in the roles of supporters, performing through acts such as bringing coffee and food (Aakeson, 2021; Abel, 2015; Amant, 2018; Dreesen, 2006; Preuss, 2005; Stark, 1993; Steiner, 1976; Wagelin-Challis, 2007), motivating older adults to getting outdoors (Dreesen, 2006; Shepherd, 2022), assisting with personal care (Amant, 2018; Rosenbeck and Thormann, 2009; Shepherd, 2022; Steiner, 1976), aiding with transportation and transfer (Rørvik, 2015; Shepherd, 2022; Wagelin-Challis, 2007), assisting with activities such as playing music (Madsen, 2020), observing and guarding older adults (Aakeson, 2022; Zavrël, 1094), housekeeping and laundry (Wagelin-Challis, 2007), and terminal care (Haller, 2021; Rosenbeck and Thormann, 2009; Stark, 1993). Furthermore, professionals were presented as familiar with older adults’ specific preferences, as exemplified with *“Sometimes Grandma prefers them [cakes] with chocolate. Her carers already know that”* (Shepherd, 2022, p. 13). The older adults performed as passive recipients of the services offered to them front stage in the area in the nursing home and backstage in the private space of their rooms. Older adults living in nursing homes were also depicted as lonely (e.g., Stark, 1993), partly because of deceased family, partly due to illness that limited their mobility (Abel, 2015; Steiner, 1976), which also made them dependent on volunteers who took on the roles of friendly visitors, as illustrated by Abel (2015). The picture books often presented older adults in a living room, with seemingly minimal interaction between the older adults living in the same nursing home, acting as a tacit audience to each other’s front stage lives and presence (Aakeson, 2022; Preuss, 2005; Madsen, 2020; Stark, 1993).

Nursing homes were also depicted as places from where older adults escaped with the help from children (Aakeson, 2022; Moen, 1988; Zavrël, 1094). Aakeson (2022) illustrated this as the doorbell rang while the grandchild was home alone. The grandmother stood outside, having escaped from the nursing home. They sat together in the garden, drank ice water, and shared life stories and fantasies (Aakeson, 2022). Older adults were also portrayed in the role of escapees with the aim to help children to live a better life away from the city’s and modernity’s tyranny of time (Bourgeat, 1984). Bourgeat (1984) illustrated how older adults left the nursing home and ventured into the “real” world to discover a new reality together with a child who had enlightened them about the new world. Their journey involved finding all children and, together with them, recreating the “good old” world where they could live in harmony, embrace simple living, slowness and time for one another (Bourgeat, 1984). One book showed nursing homes’ backstage as having no room for all old adults in need of them, which involuntarily forced older adults to live at family’s house, against the wishes of both older adults and their families (Scherfig, 2015).

In some picture books, nursing homes were presented as places of forced residence for older adults per se (Zavrël, 1094), for older adults with dementia (Aakeson, 2022; Moen, 1988) and for older adults whose houses, despite displaying resistance, were demolished due to political urban renewal initiatives (Marsoli, 2009). Zavrël (1094) illustrated nursing homes as places of forced residence for older adults as follows:

“*One day, the mayor woke up in a bad mood and decreed that all older adults in the city must relocate to a brand-new, large facility. The mayor viewed older adults as setting a poor example, no longer appearing presentable or contributing to society. They wandered the streets heedless of traffic, gave children chocolate instead of letting them watch television, and told bedtime stories at night. Relocating them to a nursing home was seen as a solution to this perceived societal issue*.”

Moen (1988), for instance, demonstrated that older people’s children and their children’s spouses could take on the decision-maker role to move them to a nursing home despite their wishes due to their senility, confusion, fatigue and childlike eating habits. Some healthcare professionals took on the role of disciplinarian also forcing older adults to stay at nursing homes. This was illustrated by Aakeson (2022) describing a front stage scenario where visitors were about to leave and the older adult wanted to go with them, and the healthcare professional exclaimed, “*No, no, you need to stay here!* […] *We’re just saying goodbye to your family! It’s almost time to eat.*”

#### Associations between nursing homes and kindergartens

Many of the scenarios in picture books evoked associations with kindergartens, reflecting Goffman’s concept of framing. The example above (Aakeson, 2022) could also reflect a farewell situation at a kindergarten where parents had to leave their child who was unhappy, they must stay at school rather than with their family. Some books explicitly described nursing homes as resembling kindergartens, such as “The Children’s House” (Bourgeat, 1984), while others portrayed professionals treating older adults like children (e.g., Aakeson, 2022; Madsen, 2020). Nursing homes were framed as controlled environments where older individuals were either “dropped off” by the middle-aged generation, usually the adult children (Moen, 1988; Shepherd, 2022; Zavrël, 1094) or were “picked up” from (or “rescued”) by children (Bourgeat, 1984; Marsoli, 2009; Moen, 1988; Stark, 1993; Zavrël, 1094). They were also a place for eating and playing (Amant, 2018; Madsen, 2020; Shepherd, 2022). Some books used vibrant colours like stark blue, green, yellow, and red (Amant, 2018; Madsen, 2020; Rørvik, 2015), mirroring kindergartens’ visual language and presenting the nursing home as a lively and engaging place. However, some books illustrated nursing homes as initially unsettling for children who viewed older adults as strange due to visible signs of ageing like white hair, wrinkles, and mobility aids (Abel, 2015; Amant, 2018; Madsen, 2020; Preuss, 2005; Wagelin-Challis, 2007). This reflected the performance of ageing, where these visual markers functioned as props that emphasised older adults’ institutionalised roles. Their lived lives further influenced their performance as older adults (Fox, 1986; Preuss, 2005; Rørvik, 2015). However, through interaction, children reframed their perception, transforming the nursing home into a more familiar and engaging space (Aakeson, 2022; Madsen, 2020; Preuss, 2005). The depiction of children getting to know the older residents and participating in play with them (Aakeson, 2022; Abel, 2015; Madsen, 2020; Moen, 1988; Preuss, 2005; Shepherd, 2022) illustrated how engagement could overcome unfamiliarity, like how children gradually adjust to new environments such as kindergartens.

Front stage, some books presented professionals in white uniforms administering medication (Amant, 2018) and watching over older adults (Aakeson, 2022; Shepherd, 2022; Stark, 1993), others depicted them as motherly and caring (Amant, 2018; Rosenbeck and Thormann, 2009; Shepherd, 2022), hospitable and cheerful (Amant, 2018; Madsen, 2020; Shepherd, 2022), playful (Madsen, 2020), or as disciplinarians (Aakeson, 2022; Zavrël, 1094), akin to kindergarten teachers. Middle-generation adults, typically children’s parents, were shown making decisions for older adults, such as placing them in care or regulating their daily routines (Lundgren, 1979; Moen, 1988; Shepherd, 2022). Meanwhile, children assumed an authoritative role and were often depicted as enabling older adults to step outside the institution’s confines (Bourgeat, 1984; Moen, 1988; Stark, 1993; Zavrël, 1094). Older adults adjusted their behaviour based on their social context or setting by acting passively with professionals and middle-generation and becoming more playful and engaged with children (Dreesen, 2006; Madsen, 2020; Shepherd, 2022; Stark, 1993; Wagelin-Challis, 2007). When interacting with children, older adults were depicted singing and dancing (Dreesen, 2006; Madsen, 2020; Wagelin-Challis, 2007), playing instruments (Madsen, 2020; Stark, 1993; Wagelin-Challis, 2007), reading, playing cards or puzzles, engaging in creative activities (Shepherd, 2022; Steiner, 1976; Wagelin-Challis, 2007) or doing outdoors activities such as walking (Lundgren, 1979), planting, gardening (Shepherd, 2022) and playing football or other physical activities (Dreesen, 2006; Fox, 1986; Stark, 1993). Such interactions happened both backstage at the room of older adults without any audience (Nivaa, 2018; Shepherd, 2022; Wagelin-Challis, 2007), and front stage in both indoor and outdoor settings, where professionals, parents and other older adults acted as audience (Dreesen, 2006; Shepherd, 2022) or co-actors (Amant, 2018; Madsen, 2020; Rørvik, 2015). The play fostered bonding and joy was conveyed textually and visually in the books (Dreesen, 2006; Madsen, 2020; Nivaa, 2018; Shepherd, 2022; Wagelin-Challis, 2007; Zavrël, 1094). These interactions presented children and older adults as peers, emphasising similarities, such as shared values and humour (Aakeson, 2022; Abel, 2015; Bourgeat, 1984; Dreesen, 2006; Lundgren, 1979; Madsen, 2020; Marsoli, 2009; Moen, 1988; Nivaa, 2018; Preuss, 2005; Shepherd, 2022; Stark, 1993; Steiner, 1976; Zavrël, 1094).

Eating and drinking played a crucial role in many books. Older adults and children were often depicted sharing cookies and coffee or juice (Aakeson, 2021; Abel, 2015; Amant, 2018; Dreesen, 2006; Madsen, 2020; Shepherd, 2022; Stark, 1993). The portrayal of these situations varied across books, but some emphasised the difficulties older adults face in eating “correctly,” drawing parallels to children who spill food or need assistance. The inability to eat properly was depicted as a sign of lost bodily control (Aakeson, 2021; Amant, 2018). Some books explicitly connected messy eating to the need for institutionalisation, implying that difficulty in eating appropriately could justify relocation to a nursing home (Moen, 1988; Preuss, 2005). In contrast, humour in these depictions softened the stigma, presenting spilling food as something normal experienced by both older adults and children, as illustrated in Amant (2018): “*Mum laughs: ‘You two.’ She wipes the stains away with a napkin*” (pp. 21–22).

Children also played a role in keeping older adults physically active. They are shown dancing, running, roller-skating, flying a kite, or climbing trees together (Amant, 2018; Shepherd, 2022; Stark, 1993; Wagelin-Challis, 2007; Zavrël, 1094). These depictions presented older adults as playful and depicted nursing homes as settings for intergenerational engagement and playfulness. For example, in Wagelin-Challis (2007), a grandfather who, despite being nearly 100 years old, still insists on racing his grandchild to the dining hall. Nursing homes thus emerged as relational spaces where meaningful friendships between older adults and children could flourish. While the older residents and children formed and nurtured their friendships, the middle generation (i.e., parents, professionals) were portrayed as bridge-builders in this process (Abel, 2015; Dreesen, 2006; Madsen, 2020; Shepherd, 2022). For instance, Abel (2015) and Madsen (2020) narrated that a girl’s mother invited her to visit a nursing home, allowing her to form a warm and caring friendship with an older adult. Some books further emphasised the uniqueness of intergenerational friendships, depicting them as genuine and mutually enriching (Abel, 2015; Bourgeat, 1984; Dreesen, 2006; Fox, 1986; Lundgren, 1979; Marsoli, 2009; Nivaa, 2018; Stark, 1993; Zavrël, 1094). These relationships aligned with a front stage setting, where the nursing home was framed as a positive space for meaningful connections (Abel, 2015; Fox, 1986; Madsen, 2020; Stark, 1993). This idea was reinforced through moments of mutual recognition, for instance: “*Morten had the best connection with Miss Nancy. They understood each other, and their names were equally long. He could tell her secrets, and he did”* (Fox, 1986).

#### The present was the past and death was the future

It was common for older adults to live in the present through memories of the past in many of the picture books. Certain present behaviours were attributed to past youthful behaviours. For example, Shepherd (2022) depicted that grandma loved to dress up nicely just like when she was young, and Lundgren (1979) portrayed a grandpa still hiding tobacco pouches in a flowerpot like he did when he was youthful. In Preuss (2005), backstage in the older adults’ private room without an “adult” audience, the grandfather told his grandson stories about activities that the other residents experienced in their past by incorporating other nursing home settings (e.g., the corridor, dining area, and room) and props (e.g., feeding tubes, broccoli, cup of water). In several picture books, the children had the role as bridge-builders between the older adults’ past and present, while the other adults were rarely present or involved in these interactions. The children prompted and evoked these stories through conversation or by stimulating memories by using props as a reminder (Aakeson, 2022; Fox, 1986; Lundgren, 1979; Madsen, 2020; Nivaa, 2018; Preuss, 2005; Shepherd, 2022; Zavrël, 1094). Often these interactions were set backstage in the older adults’ nursing home room, and props included items in a handbag (Nivaa, 2018, e.g., comb, watch, broken lipstick), a football (Fox, 1986), and an accordion (Wagelin-Challis, 2007). Older adults with dementia or cognitive decline were shown as not connecting to the present and forgetting the past, which left them in a state of absence even though they were physically present (Aakeson, 2021, 2022; Dreesen, 2006; Moen, 1988; Scherfig, 2015). This depiction also silenced these characters in the present, as they were explained as having forgotten key attributes of their life (Shepherd, 2022), such as their birthday (Lundgren, 1979), where they had lived previously (Aakeson, 2022) or their children (Dreesen, 2006). As reminders, the nursing home rooms were often decorated with framed pictures of people and places from the past (Haller, 2021; Lundgren, 1979; Stark, 1993). In front stage spaces such as in living rooms, the older adults were depicted alone, frequently shown sitting in a chair, engaged in activities such as knitting (Stark, 1993), reading a newspaper (Stark, 1993), drinking tea (Aakeson, 2022), or simply staring at no discernible object (Aakeson, 2021; Moen, 1988; Rørvik, 2015). These portrayals indicated that the activities that counted as “living” had already happened in the past. The present time in older age was used to think back to the past rather than continue living for the future (Fox, 1986; Nivaa, 2018; Shepherd, 2022). Signs of minor cognitive impairment were also shown through messy rooms with personal items scattered around (Aakeson, 2022; Wagelin-Challis, 2007) and forgetting important dates (e.g., one’s own birthday) (Lundgren, 1979; Stark, 1993) and visitation agreements (Amant, 2018; Stark, 1993), which enforced the message that older people who got confused or forgetful need the help provided at a nursing home (Shepherd, 2022; Steiner, 1976). Older adults were only depicted as active in the present and having a future in few books (Bourgeat, 1984; Rosenbeck and Thormann, 2009; Zavrël, 1094). Rosenbeck and Thormann (2009) portrayed a grandfather who was admitted to a nursing home following a stroke yet still maintained cognitive clarity which created dissonance between his perceived alertness and the need for nursing care. Despite the stroke, he was still engaged in reading the newspaper, listening to the radio, and watching television, which indicated he was still intellectually active just as he was prior to the stroke. In two other books where the older adults moved away from the nursing homes, the older adults were portrayed as active, enterprising citizens that worked with the children to be freed from the nursing home (Zavrël, 1094) or saved the children from the modern, stressful world and created a better world for them that resembled the childhood life of the older adults (Bourgeat, 1984).

There were a few depictions of the future that focused on inevitable death. The books often showed a romanticised and idealised depiction of dying and death illustrated by a peaceful and calm process, often surrounded by close, beloved family members backstage in the older adults’ room (Haller, 2021; Rosenbeck and Thormann, 2009; Stark, 1993). Death happened quickly following an acute illness event (e.g., stroke, heart issue) (Rosenbeck and Thormann, 2009; Stark, 1993) or simply due to old age (Haller, 2021; Wagelin-Challis, 2007) resulting in a swift, peaceful passing. Death was shown with the older person tucked cosily in a hospital bed surrounded by adult children, grandchildren, and extended family members and framed photos of the past (Haller, 2021), with a nuclear family around the deceased in a hospital bed with candles and flowers (Haller, 2021; Rosenbeck and Thormann, 2009), with a neatly-made but empty bed in an empty room (Stark, 1993), or not shown at all, with the older person becoming absent from the story until death is revealed to the children at the funeral (Stark, 1993; Wagelin-Challis, 2007). Stark (1993) presented the nursing home located next to a church, as if to show that following a stay at the nursing home, the future was in a grave. In several books, nursing homes were described in a way that can be interpreted as a waiting room for death, as a place that precedes death; which was illustrated through lonely older adults dressed in dark clothing sitting together in common areas in silence and not interacting with each other, barely moving, and staring into space (Aakeson, 2021; Abel, 2015; Moen, 1988; Stark, 1993). In contrast, Preuss (2005) showed that some of the older adults in the living room were socialising and talking with each other. Other books explained that nursing homes were a place for those who have age-related disabilities that are contributing to their decline (Amant, 2018; Preuss, 2005), signalling that an inevitable death was in the near future. For many of the older adults characterised in these stories, they believed “real life” was outside the nursing home, and therefore their real life already happened in the past (Aakeson, 2022; Bourgeat, 1984; Marsoli, 2009; Moen, 1988; Stark, 1993; Zavrël, 1094), and their future was death (Haller, 2021; Rosenbeck and Thormann, 2009; Stark, 1993).

## Discussion

The discussion will focus on three main findings: (1) a prevailing narrative highlighting the positive aspects of daily life in nursing homes for older adults, (2) the concept of intergenerationality under threat; and (3) a romanticised view of death alongside an ideal family structure. Finally, the strengths and limitations of this study will also be discussed.

The findings indicate a dominant narrative concerning the positive everyday life in nursing homes for older adults, with associations drawn to the positive experiences of kindergarten life. This stood in contrast to other presented narratives that described these places as forced residences, locations from which older adults escaped from, and places lacking capacity for all old adults in need. Different types of older adults were related to those narratives. Crăciun and Făgărăşan (2020) illustrate how Romanian children aged 11–14 perceive older adults through various stereotypes. These include the *friendly grandmother* (caring and domestic), the *rebellious grandmother* (defying tradition), the *psychopathic older man* (frightening and dangerous), the *weak and frail elderly* (helpless and dependent), and the *wise elder* (knowledgeable and respected). These stereotypes reflect a spectrum of attitudes toward ageing, encompassing both positive and negative perceptions. Our findings also revealed stereotypes surrounding older adults, with some of these stereotypes presented as positive and nuanced portrayals. For instance, older adults are depicted as kind, playful, and storytellers. Positive representations can foster empathy, mitigate ageist attitudes, and enhance intergenerational understanding. Employing a presentation style that encourages readers to perceive nursing homes as analogous to other well-known institutions, such as kindergartens, can facilitate children’s ability to relate to their own institutional life. However, the results also revealed that the picture books portrayed a limited racial and ethnic diversity among older adults, predominantly depicting them as white people, which contrasts with the diversity found in many Danish kindergartens today (Dannesboe and Kjær, 2021).

Older adults in nursing homes can be compared to children in kindergartens due to their reliance on caregivers for daily activities, their structured routines, and the emphasis on social interaction to prevent social isolation. Cognitive and physical decline, particularly in those living with dementia, may result in behaviours resembling those of children in emotional expression and understanding. Furthermore, both settings utilise play and activities to promote mental and physical engagement, requiring supervision to ensure safety. Although nursing homes are often portrayed in societal discourse as medicalised institutions where older adults experience decline, these picture books offer an alternative perspective—one that emphasises relationships, intergenerational bonds, and playfulness. This viewpoint challenges prevailing narratives of ageing as a process of isolation and decline. However, it also risks oversimplification by minimising elder care’s complexities and institutional constraints. Moreover, it might suggest an infantilisation where older adults residing at nursing homes are not represented as “real adults,” but rather akin to children. Infantilisation of older adults in nursing homes is also shown in research (Castle et al., 2015; Zhao et al., 2021). As demonstrated in our findings, children and older adults were sometimes depicted as equal playmates, while in others, the children appeared more “adult-like” than the older adults, such as when they helped their grandparents remember things through different games. Meanwhile, stereotypical or negative portrayals might inadvertently reinforce fear or misunderstanding regarding older adults and institutional care. In light of Goffman (1986), the findings also illustrated a stigmatisation of older adults in nursing homes, as these institutions are associated with loss of autonomy, physical decline, and dependency. Marson and Powell (2014) explore whether infantilisation is a form of care or a sign of disrespect, highlighting that older adults in nursing homes are frequently pitied and treated as less competent. This observation aligns with Jongsma and Schweda (2018), who argue that the perception of dementia as a return to childhood can lead to a flawed understanding of dementia, thereby influencing our attitudes and behaviours toward those affected in problematic ways. Exposing children to books featuring a diverse range of older characters can hence foster a more nuanced understanding of age and older individuals (Larkin et al., 2013).

The current findings highlight the dependence of older residents in nursing homes for daily personal and medical needs. Marson and Powell (2014) show that older adults are often perceived as helpless and spoken to in a condescending, patronising manner in nursing homes. Their study suggests that, to avoid conflict with caregivers, some residents may assume the role of *cynical actors* (Goffman, 1990), passively accepting infantilisation out of fear that their reliance on care makes them vulnerable to neglect should they resist. As a result, when staff speak to them in a childlike manner, older residents may not withdraw or avoid cooperating as *sincere actors*, as they are genuinely committed to their performance. Instead, they may respond in ways commonly seen in total institutions, where individuals are separated from wider society for an extended period and live under strict control, by accepting their circumstances (Goffman, 1961). The picture books depicted older adults as either passively accepting this living situation or resisting it to some extent; one form of resistance involved escaping from or leaving the nursing home. Older adults may fear losing status when moving into a nursing home and attempt to escape with the help of their grandchildren, as one of the counter-images illustrates. Another counter-image highlighted the lack of room in nursing homes for all older adults in need of them. This presentation on nursing homes aligns with current western political strategies on “ageing in place” (European Commission, 2021; World Health Organization, 2015), which aim to reduce nursing home capacity (Sharma et al., 2024; Sullivan, 2024) in favour of older adults staying in their homes longer. Only three of the books displayed a socially critical stance, problematising modern values, the “tyranny” of time, and the demands of contributing to society (Bourgeat, 1984; Zavrël, 1094), as well as the lack of capacity in nursing homes (Scherfig, 2015). However, the study’s results said nothing about how children read and interpret picture books, calling for future research. From a Goffmanian perspective, child readers could be seen as the audience of the picture books themselves, adding another dimension to future research on the power and effects of socialisation conveyed through picture books in “real life.”

Furthermore, the findings indicate that the picture books depicted older adults socialising most effectively with children, while interactions with children’s parents and professionals were minimal. Older adults rarely engaged with one another in the public, front stage areas of nursing homes. Additionally, social interactions between older adults within their own rooms were not represented. In other words, the intergenerational bond between children and older adults was strong, while the middle generation became less significant in their direct relationship with older adults/their parents. However, they still played a crucial role as bridge-builders between the older and younger generations. This can be seen as children taking on a social responsibility for older adults, acting as preventers of loneliness, playmates, and memory evokers. This aligns with the concept of lifelong learning, in which children and older adults teach and learn from one another through their close relationships (UNESCO, 2022). According to UNESCO (2022), family learning focuses on intergenerational communication and supports lifelong learning. The current findings also illustrated the trend of organised intergenerational projects in nursing homes, where children visit older adults, becoming increasingly prevalent. Such initiatives are global and aim to bridge the generational gap, promote social interaction, and provide emotional and cognitive benefits for both age groups (Gualano et al., 2018; Jones and Ismail, 2022; Lyndon and Moss, 2023; Scheffel, 2015; Whear et al., 2023). For instance, Scheffel (2015) reflects on the role of grandparents in children’s lives and discusses, among other things, how books with intergenerational themes can be utilised to strengthen the connection between older adults and children. The author highlights that no single definition of the grandparents’ role exists, but intergenerational themes can help children understand more about ageing (Scheffel, 2015). However, attention is required to avoid simultaneous infantilisation of older adults, allowing children and older adults to meet on equal, genuine terms that emphasise shared experiences and a sense of community (Castle et al., 2015; Larkin et al., 2013; Zhao et al., 2021). Not all children find interacting with older adults rewarding, nor do all older adults find spending time with children fulfilling, even though several of the included picture books conveyed such a message.

A few picture books focused on the dying and death of older adults in nursing homes, contrasting with the more significant number of books addressing death in other settings, bereavement, and grief (e.g., Fu et al., 2025; McGuire et al., 2013; Wiseman, 2013; Sgier et al., 2024). In this way, the picture books indirectly support the political trends surrounding “ageing in place,” encompassing the notion that older adults die at home (Alders and Schut, 2019; Buffel and Phillipson, 2024; Finlay and Finn, 2021). Building on the study’s findings, it could be suggested that the picture books may imply that the loss of abilities, and more broadly, moving to a nursing home, may signal the approach of death. The included books often presented a romanticised view of dying and death of older adults in nursing homes. None of the included picture books addressed themes of sudden or violent death but were in line with the more common portrayal of death as a peaceful and natural conclusion (Kivits, 2022). This pattern aligns with findings from a Chinese study on picture books (Fu et al., 2025), a Swedish study on storybooks for children aged 6–12 (Eklund, 2024) and a US study on picture books (Danielson and Colman, 2023), all highlighting a societal preference for romanticised depictions of death. Such portrayals influence how children internalise the concept of loss. While shielding children from distressing narratives may offer emotional protection, it can also limit their ability to process real-life experiences of death (Romero, 1976). Literature that openly acknowledges grief and bereavement can provide valuable emotional support, aiding children in navigating their encounters with loss (Danielson and Colman, 2023; Eklund, 2024). Overly idealised representations of death and mourning may leave children unprepared for more difficult experiences, such as those resulting from serious illness or accidents (Arruda-Colli et al., 2017; Schonfeld, 1993). Additionally, the use of euphemisms or the omission of harsh realities can foster confusion rather than clarity, particularly for children facing death in their own lives (Arruda-Colli et al., 2017). Furthermore, the idealised portrayal of families concerning dying older adults ignores the complexities of familial dynamics, including geographical distance, strained relationships, illness, conflicting responsibilities, and various types of families, both with children and those without family (Gulestø et al., 2022; Oute and Glasdam, 2022; Vedsegaard and Wind, 2024). The picture books predominantly portrayed white, middle-class families, and heteronormative family structures. The underrepresentation of non-traditional family structures may reinforce the notion that the “traditional” nuclear family—comprising a mother, father, and children—remains the societal norm, while alternative family forms are either erased or pushed to the margins (Mokrzycki, 2020). Statistics Denmark (2018) has identified 37 different types of families with children in Denmark. These limited portrayals may contribute to a narrow definition of what children consider “normal,” particularly when the literature also excludes diverse racial, ethnic, and socioeconomic backgrounds (Bland, 2016; Leahy and Foley, 2018). A more inclusive approach is essential to challenge these norms and foster a future generation that values inclusion, diversity, and intergenerationality. This calls for a broader and more nuanced understanding of family in future picture books for children.

The study’s method has strengths and limitations. The searches were carried out systematically, following the study’s inclusion and exclusion criteria, and spanned a 50-year period, focusing on picture books for preschool children, which strengthened the study’s credibility and dependability (Lincoln and Guba, 1984). This approach resulted in a modest sample of 22 books from nine countries. However, only those available in Danish public libraries were included. It is worth noting that other relevant picture books meeting the inclusion criteria may exist in the Danish market but were inaccessible through the library system, and consequently, they were not included in this study. Hereby, the results’ transferability may be challenged (Lincoln and Guba, 1984); however, other studies show that many children’s picture books are translated into various languages and are available to children in many countries (Glasdam et al., 2024a; Fu et al., 2025). The three authors held ongoing discussions throughout the process of including and excluding books, contributing to the study’s trustworthiness. All authors actively participated in the analysis, continually engaging with the empirical material—both images and texts—while developing and refining themes. This iterative process ensured fidelity to the empirical material, thereby enhancing the study’s credibility and confirmability (Lincoln and Guba, 1984). Furthermore, the authors consistently reflected on Goffman’s theoretical framework and the analytical questions throughout the entire analytical process, ensuring alignment with the study’s aim and the constructed themes. This approach enabled the authors to apply thematic analysis in a deliberate, informed, and reflexive manner, as recommended by Braun and Clarke (2020). Through its clear theoretical framework, this study allows the reader to follow the analysis and gain insights drawn from this specific perspective. The study’s strength lies in its transparent analytical approach, enabling readers to witness the construction of “truths” about the analysed picture books. Additionally, the theoretical framework helped the authors transcend their potential preconceptions (Glasdam et al., 2024b), which also strengthened the study’s trustworthiness.

## Conclusion

The study showed that nursing homes were generally depicted as institutions that provide care for older adults who have reached a point in their lives due to age or illness where they are no longer independent. Nursing homes were also shown as places of forced residence, places to escape from, and places without room for all old adults despite their needs. Many of the picture books created parallels between older adults in nursing homes with multiple associations to kindergarten that were recognisable to children as readers. At the same time, there was a tendency toward the infantilisation of older adults within this representation. There was a relatively equal relationship between children and older adults, while the middle generation functioned as a bridge between the outer generations without much active interaction with older adults. Relatively few books dealt with the death of older adults in nursing homes, but those that did presented a romanticised notion of the end of life, where the mourning family was also depicted as an ideal unit who dealt with grief solemnly. Future research on this topic is needed to explore children’s interpretations of the picture books and whether and how they influence perceptions of nursing homes and the older adults living in them. Families are becoming increasingly diverse, and while we only looked at sex and age-related attributes of the picture book characters in our analysis, future research in this area could explore the characteristics of families in picture books and how they are reflected in the current family units, not only in Denmark, but also generally in the western world. Furthermore, the limited portrayals of end-of-life and death highlights the need for future research into whether existing children’s picture books address this topic and how they might adopt more pragmatic or realistic approaches to presenting end-of-life and death to young readers.

## Data Availability

The original contributions presented in the study are included in the article/supplementary material, further inquiries can be directed to the corresponding author.

## Footnotes

^1^ https://www.odensebib.dk/

^2^ https://bibliotek.dk/
